# Practical Method to Evaluate the Stiffness of Fractured Radius

**DOI:** 10.3390/mps6030056

**Published:** 2023-06-05

**Authors:** Nitikorn Noraphaiphipaksa, Surangkana Katepun, Thanapong Waitayawinyu, Chaosuan Kanchanomai

**Affiliations:** 1 Department of Mechanical Engineering, Faculty of Engineering, Thammasat School of Engineering, Thammasat University, Klong Luang 12120, Pathumthani, Thailand; 2 Department of Orthopaedics, Faculty of Medicine, Thammasat University, Klong Luang 12120, Pathumthani, Thailand

**Keywords:** radius, biomechanical test, biomechanical apparatus, stiffness

## Abstract

Distal radius fractures (DRFs) are one of the most common fractures of the upper extremity system. To evaluate the performance of DRF treatments, the construct (i.e., a DRF fixed by an implant) was compressed at the distal radius in the axial direction to evaluate the compressive stiffness. In previous studies, various constructs of both cadaveric and synthetic radii have been proposed for biomechanical testing for DRF. Unfortunately, high deviations of the measured stiffness have been reported across the literature, which may relate to the inconsistency of applied mechanical actions (i.e., the tested radii may under various combinations including compression, bending, and shear). In the present study, a biomechanical apparatus and an experimental procedure were proposed for the biomechanical testing of radii under pure compression. After the biomechanical tests of synthetic radii, it was found that the standard deviation of stiffness was significantly lower than that in previous studies. Thus, the biomechanical apparatus and the experimental procedure were proven to be a practical method for the evaluation of radii stiffness.

## 1. Introduction

Distal radius fractures (DRFs) are one of the most common fractures of the upper extremity system. In the United States, DRFs comprise approximately 20% of cases treated at emergency medicine departments [1]. As a medical device to support a fractured bone, metal implants are used for the treatment of DRFs. The micromotion, misalignment, and overload of a DRF fixed by implant may cause severe deformation, and subsequently lead to fracture of the implant [1]. To evaluate the performance of a DRF treatment, biomechanical tests are usually performed on a construct, composed of a DRF fixed by implant. The construct is cyclically compressed at the distal radius in the axial direction to simulate the loading during a healing period and/or a physical therapy period [2,3,4]. As the parameters showing the performance of construct, the stiffness and the load-to-failure are determined from the biomechanical test. The stiffness of a construct is the slope of the initial linear part of the relationship between the compressive force and the deformation of the construct. After the completion of cyclic compression, the construct is compressed until failure, where the load-to-failure is evaluated. Notably, the objective of this biomechanical test is different from directly subjecting an implant (e.g., a distal radius plate) to a mechanical test, where the performance of an implant is specifically evaluated.

Biomechanical tests using a cadaveric radius can provide the actual interaction between implant and DRF; therefore, the cadaveric radius has been used for biomechanical tests. Unfortunately, the high variabilities in geometry and material properties of cadaveric radii can cause significantly high deviations of stiffness [5,6,7]. To avoid high variabilities in geometry and material properties, as well as to accommodate the special handling requirements of cadaver radii, the synthetic radius was designed to simulate structural and material properties of cadaveric radius, while providing the low variabilities in geometry and material properties, as well as fulfilling the simple handling requirements. Synthetic radii have frequently been used for the biomechanical tests of treated DRFs [8,9,10]. Although the variabilities in geometry and material properties of synthetic radii can be controlled, the high deviations of stiffness are surprisingly observed in these studies. The high deviations make it difficult for comparisons between various DRF treatment methods to be performed.

During biomechanical tests, pure compression without any bending and/or shear is expected. It is speculated that the high deviations of stiffness [8,9,10] may relate to the inconsistency of applied mechanical actions (i.e., the tested synthetic radii may be under the various combinations between compression, bending, and shear). In the present study, a biomechanical apparatus and an experimental procedure were proposed for the biomechanical tests of radii under pure compression. The biomechanical apparatus and the experimental method were used for repeated biomechanical tests of synthetic radii. As a statistic that represents the dispersion of data from its mean value, the standard deviation (SD) of stiffness was determined, and compared with those of previous studies. Subsequently, the applicability of a biomechanical apparatus and experimental method for the biomechanical tests of synthetic radii was discussed.

## 2. Materials and Methods

### 2.1. Synthetic Radius

As an alternative to cadaver bone, synthetic bones (i.e., Sawbones: composite bones for test and validation studies) have been designed to simulate structural and material properties of cadaveric bones. Because they are made of composite materials, the handling requirements of synthetic bones are minimized, and their material properties can be maintained over a long period of time. The variabilities in geometry and material properties among various pieces of synthetic bone are also strictly controlled by the manufacturer. Accordingly, they are commonly used for the comparative and developmental testing of orthopedic devices and instrumentation.

In the present study, a synthetic radius was used as a specimen to investigate the applicability of a new biomechanical apparatus for the biomechanical test. The synthetic radius was the fourth-generation composite left radius (Sawbones, a Pacific research company, Vashon, WA, USA: Model 3407). According to the manufacturer, the cortical shell is made of short fiber-filled epoxy, while the cancellous core is made of solid rigid polyurethane foam (i.e., 17 pounds per cubic foot or 272.3 kg per cubic meter). The cortical bone has a compressive modulus of 16.7 GPa and a compressive strength of 157 MPa. Meanwhile, the cancellous bone has a compressive modulus of 0.155 GPa and a compressive strength of 6 MPa.

### 2.2. Design of Biomechanical Apparatus

To generate pure compressive force during the biomechanical test, applied compressive force at the distal radius and reaction compressive force at the proximal radius were placed on a reference line which passed through the mass center of radius. Accordingly, the center line of the fixture at the proximal radius and the center line of the applied loading device at distal radius were aligned with the reference line of radius. The details of biomechanical apparatus are described as follows.

Reference line of radius

The synthetic radius was scanned using a laser scanner (Faro Laser ScanArm, Lake Mary, FL, USA: Edge) and modeled in commercial FEA software (i.e., Abaqus/Standard [11]). The 44 mm distal radius and 43 mm proximal radius were removed from the FE model, and the 158 mm of remaining shaft was equally divided into 16 sections. The mass centers of these sections were determined and averaged; subsequently, a reference line was created through the averaged mass center.

Fixture at proximal radius

**Figure 1:**
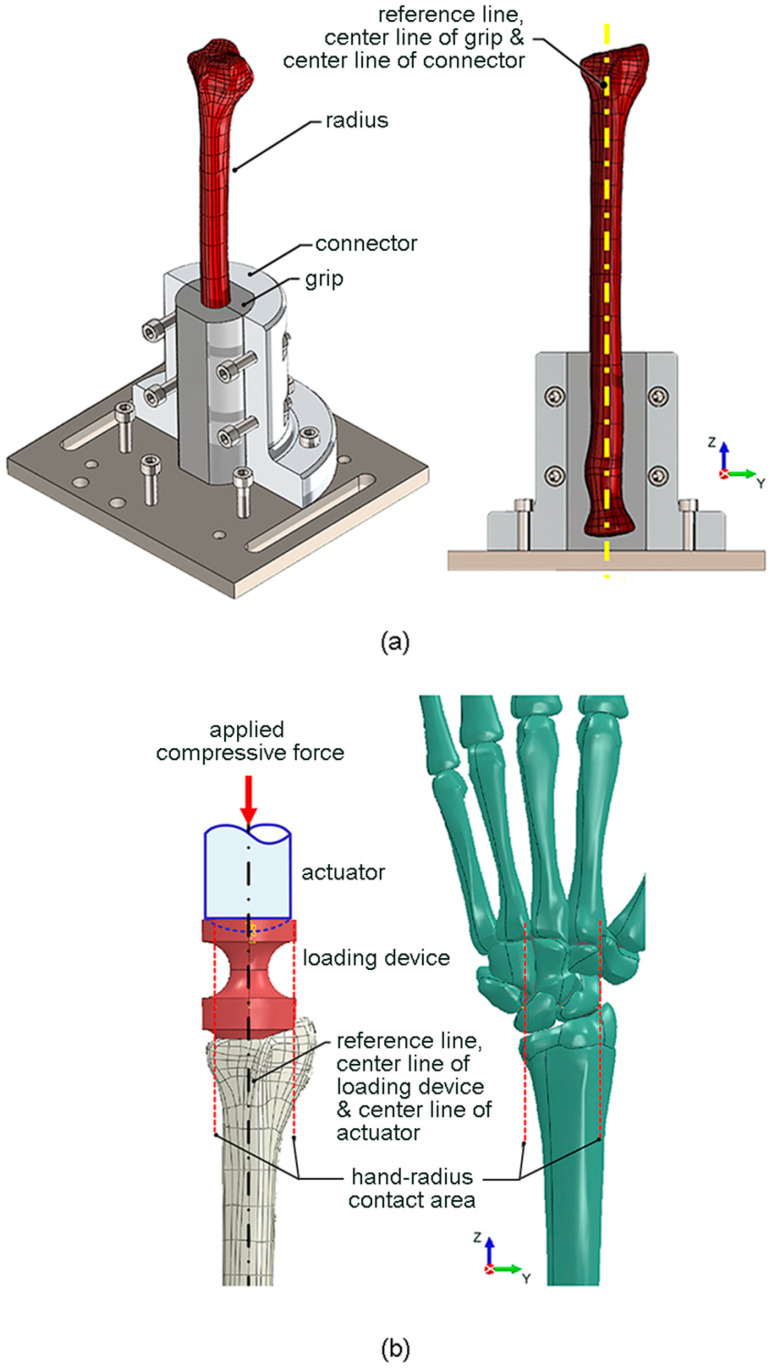
(**a**) Fixture at proximal radius; (**b**) loading device at distal radius.

To securely fix the proximal radius to the testing machine, a special fixture (i.e., a grip and a connector) was designed, as shown in Figure 1a. The grip had the inner contour similar to the outer contour of the proximal radius (i.e., a scan profile of synthetic radius), and the center line of the grip was aligned with the reference line of the radius. The grip was connected to the testing machine via a connector, with its center line aligned with the center line of grip and the reference line of radius. The proximal radius was tight-fitted by the grip, and the connector was fixed to the testing machine using bolts. The grip was 3D-printed using an engineering epoxy, which had an elastic modulus of 675 MPa, and a tensile strength of 54 MPa. On the other hand, the connector was made of 6063 aluminium alloy, which had an elastic modulus of 68 GPa and a yield strength of 214 MPa.

Loading device at distal radius

Edwards and Troy [12] reported that the application of compressive force directly to the isolated radius underestimated the mechanical importance of the trabecular compartment compared with the more physiologically relevant intact wrist scenario (i.e., the application of compressive force through the hand with the wrist joint intact). Therefore, in the present study, a special loading device was designed to simulate the compressive force between the wrist joint and the distal radius (i.e., a scan profile of synthetic radius), as shown in Figure 1b. The lower contour of the loading device was designed to represent the contact surface between the wrist joint and the distal radius. The centroid of the contact surface was aligned with the center line of the loading device and the reference line of the radius. The compressive force was applied by a spherical-end actuator of the testing machine, with its center line also aligned with the center line of the loading device and the reference line of the radius. The loading device was 3D-printed using an engineering epoxy, while the spherical-end actuator was composed of 6063 aluminium alloy.

Stress distributions on radius and biomechanical apparatus

To accurately perform the biomechanical test, the stress distribution around the distal radius should not be influenced by the fixture at proximal radius. Thus, the stress distributions of case i (i.e., an intact radius which was gripped around the 93 mm part of the proximal radius) and case ii (i.e., a sectioned radius in which the proximal radius was removed) were numerically calculated and compared.

During the fracture healing period for a patient, the compressive force on a treated DRF is unlikely to be greater than 150 kgf or 1500 N. To provide confidence, a maximum compressive force of 1500 N was used for the numerical investigations of the influence of the fixture at the proximal radius on the stress distribution around distal radius, as well as the strength of the new biomechanical apparatus. According to the preliminary FEA, the stress distributions around the distal radius based on the linear–elastic behavior and the elastic–plastic behavior were marginally different. To simplify the FEA, the FE model of the radius was assumed to have a linear–elastic behavior under the maximum compressive force of 1500 N. The radius was modeled using 4-node tetrahedral elements. The Poisson’s ratio used for the FEA was 0.45. The arbitrary sizes of element were initially assumed, and the stresses were numerically calculated. To minimize the influence of the element size, the element size was adjusted until the variation in stress was lower than 5%. There were 49,130 elements and the element size of an intact radius (i.e., case i) was 0.15 to 2 mm.

### 2.3. Biomechanical Test

To manage pain and to regain the motion, strength, and function of the radius, DRF rehabilitation is divided into 3 stages: splinting, mobilization, and strengthening [13]. During splinting, wrist range of motion (ROM) exercises are performed to increase the ROM of the digits, wrist, and forearm. During mobilization, additional ROM is given to improve the motion and the overall function, while pain and edema are controlled. During strengthening, further ROM exercises are performed to regain normal function, while reducing the level of impairment. Unfortunately, the actual force on the distal radius during DRF rehabilitation has not yet been reported.

**Figure 2:**
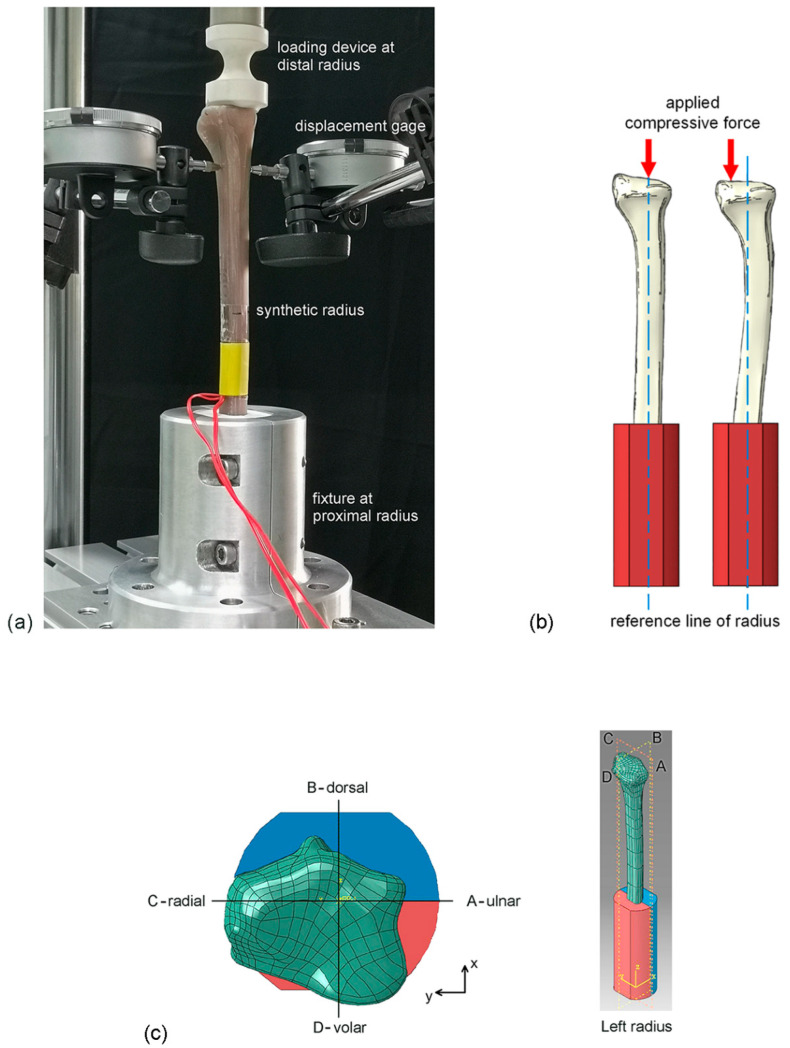
(**a**) Setup of the biomechanical test; (**b**) deflection of the distal radius in the horizontal plane; and (**c**) positions of the measurements of deflection.

The ROM of the digits, wrist, and forearm is controlled during DRF rehabilitation; thus, application of a compressive force of 10 kgf or 100 N in the axial direction of the radius was assumed during the experimental evaluation of the new biomechanical apparatus. The displacement-controlled compressive test was performed using an electromechanical testing system (Instron, Norwood, MA, USA: ElectroPuls E10000 with 5-kN load cell), as shown in Figure 2a. The synthetic radius was compressed at a displacement rate of 2.5 mm/min to the maximum compressive force of 100 N. During the compressive test, the compressive force (*F*) and the compressive displacement (*δ*) were simultaneously recorded. The stiffness of the radius was determined from the slope of the force–displacement curve (*F*–*δ* curve).

The objective of this study was to investigate the performance of a new biomechanical apparatus, and the variabilities in geometry and material properties of synthetic radius is assumed to be significantly low and can be neglected; a single synthetic radius was therefore used for the repetitive compressive tests (i.e., 10 times or *n* = 10). At the end of each repetition, the biomechanical apparatus and the synthetic radius were disassembled and removed from the electromechanical testing system. Subsequently, they were left at 25 °C for 30 min to relax any residual stresses from the previous compressive test.

During the biomechanical tests, pure compression without any bending and/or shear was expected. However, if the applied compressive force was not aligned with the reference line of the radius, the tested radius may have been under the influence of bending, and deflection of the distal radius in the horizontal plane was likely to occur (Figure 2b). To confirm the conditions of pure compressive force, deflection of the distal radius in the horizontal plane at 100 N compressive force was measured using a displacement gage with a resolution of 0.01 mm. The positions of deflection measurement (i.e., at 20 mm below the top of distal radius) are shown in Figure 2c. After 10 repetitive compressive tests, the means and the SDs of stiffness and deflection were statistically analyzed.

## 3. Results

### 3.1. Stress Distributions on the Distal Radius and Biomechanical Apparatus

**Figure 3:**
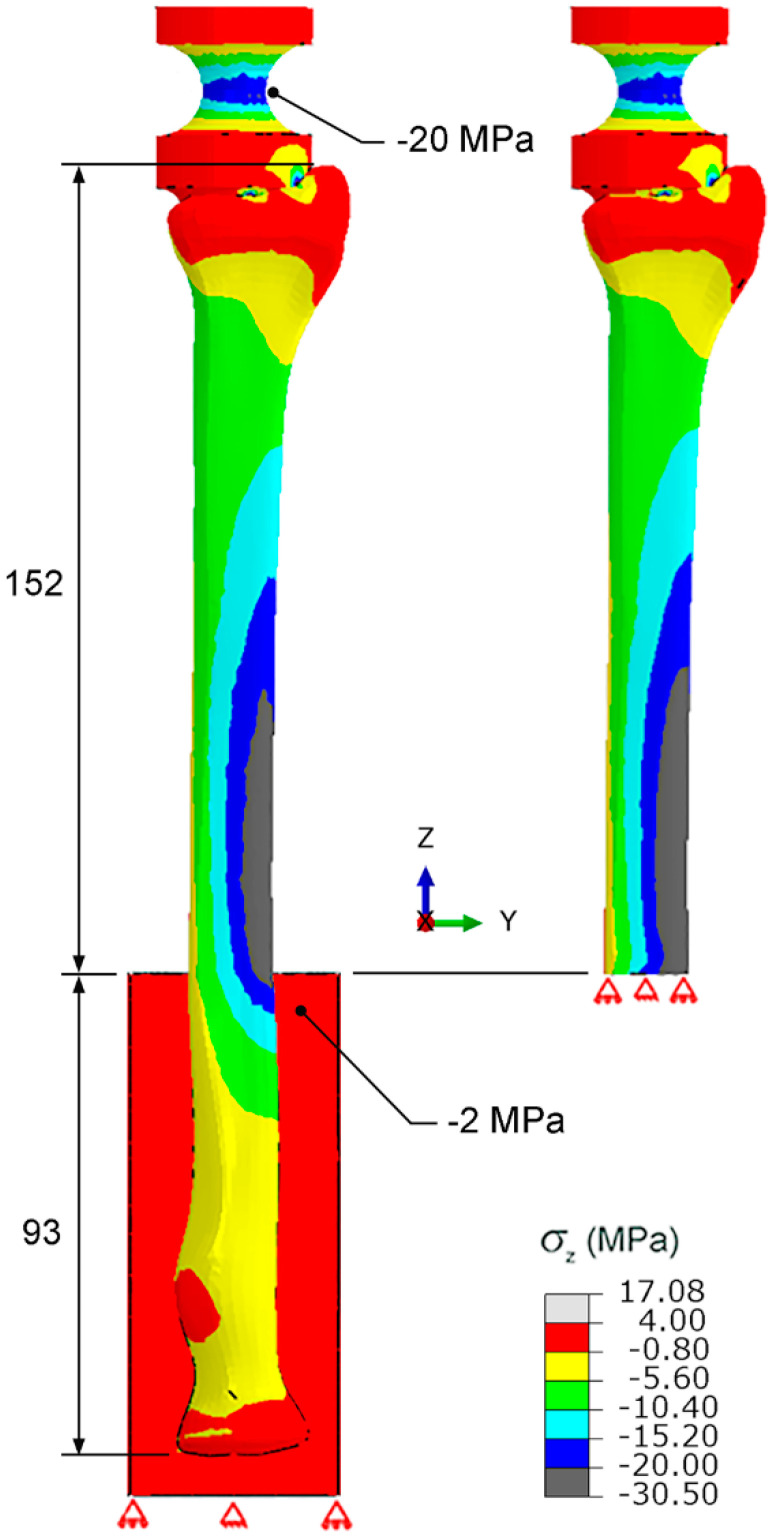
Compressive stresses on the radius and biomechanical apparatus (dimensions in mm).

The stress distributions of case i (i.e., an intact radius which was gripped around the 93 mm part of proximal radius) and case ii (i.e., a sectioned radius in which the proximal radius was removed) were numerically calculated and compared, as shown in Figure 3. The distributions of compressive stress in the *z* direction around the shaft and the distal radius of both cases are similar. Under the compressive force of 1500 N, the maximum compressive stresses on the grip of the fixture at the proximal radius and that on the loading device at the distal radius were −2 MPa and −20 MPa, respectively.

### 3.2. Stiffness of the Synthetic Radius

**Figure 4:**
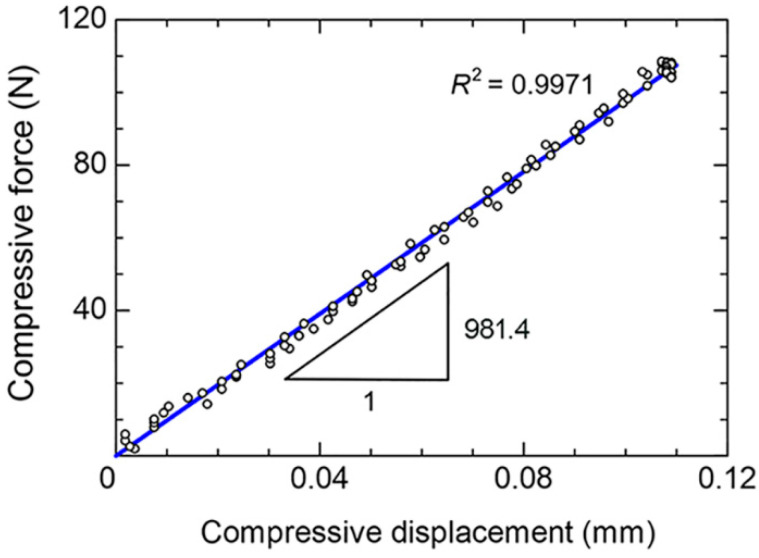
Force vs. displacement curve (*F–δ* curve).

The force–displacement curve from the biomechanical test is shown in Figure 4. As a parameter represents the quality of linear fitting, the coefficient of determination (*R*^2^) was 0.9971. Thus, the linear deformation behavior of the radius could be confirmed, and the stiffness of the radius could be determined from the slope of the *F*–*δ* curve. The stiffness values of each repetition, as well as the means and the SDs, are listed in Table 1. The mean stiffness was 1004.3 N/mm, and the SD was ±15.3 N/mm.

### 3.3. Deflection of the Distal Radius in the Horizontal Plane

The mean deflections, and the SDs are listed in Table 1. At 100 N compressive force, the mean deflections in the −*x* direction of 0.011 mm and 0.008 mm occurred on the volar and dorsal sides, respectively. On the other hand, mean deflections in the *y* direction of 0.070 mm and 0.062 mm occur on the radial and ulnar sides, respectively. The SDs of the deflections were in the range between 0 and ±0.0042 mm.

## 4. Discussion

As shown in Figure 3, the distributions of compressive stress in the *z* direction around the shaft and the distal radius of an intact radius which was gripped around the 93 mm part of proximal radius (case i) and a sectioned radius which the proximal radius was removed (case ii) were similar. Thus, the influence of the fixture at the proximal radius on the stresses around distal radius was not significant, and the fixture around the 93 mm part of the proximal radius could be used for the biomechanical test. On the other hand, the maximum stress value on the grip of the fixture at the proximal radius, and that on the loading device at the distal radius (i.e., −2 MPa and −20 MPa, respectively), were lower than the strength of an engineering epoxy (i.e., 54 MPa). Therefore, the fixture at the proximal radius and the loading device at the distal radius were strong enough to be used during the biomechanical test.

The linear deformation behavior of the radius during the biomechanical test could be determined with the coefficient of determination (*R*^2^) of 0.9971, as shown in Figure 4. Thus, the stiffness of the radius could be determined from the slope of *F*–*δ* curve. Based on 10 repetitions, the mean stiffness was 1004.3 N/mm and the SD was ±15.3 N/mm (Table 1). The low SD of the stiffness (i.e., approximately 1.5% of the mean stiffness) confirmed the repeatability of the biomechanical test using the new biomechanical apparatus.

A maximum mean deflection of 0.070 mm is observed on the radial side (Table 1). Based on the length of radius above the fixture at proximal radius (i.e., an effective length of 152 mm), the maximum deflection at the radial side was significantly small (i.e., approximately 0.05% of the effective length). Moreover, the measured deflections were nearly similar for each repetition, i.e., the SDs were in the range between 0 and ±0.0042 mm. This indicated that the conditions of pure compressive force could be achieved using the new biomechanical apparatus, which consequently caused the low variability in the measured stiffnesses.

For the biomechanical tests using cadaver radii, the variability in the measured stiffnesses could be related to the variabilities in the geometry and properties of cadaver radii and/or the biomechanical apparatus. On the other hand, for the biomechanical tests using synthetic radii, the variability in the measured stiffnesses could be related to the biomechanical apparatus. To ensure the improvement of the new biomechanical apparatus, the SD of measured stiffnesses using the new biomechanical apparatus was compared with those of previous studies (Hsiao et al. [5]; Salas et al. [6]; Yamazaki et al. [7]; Zysk and Lewis [8]; Neder Filho et al. [9]; Oh et al. [10]), as shown in Table 2. The SD of stiffnesses from the new biomechanical apparatus (i.e., ±15.3 N/mm) was significantly lower than those of previous studies, i.e., ±56 to ±112 N/mm for cadaver radii [5,6,7], and ±34 to ±274 N/mm for synthetic radii [8,9,10].

**Figure 5:**
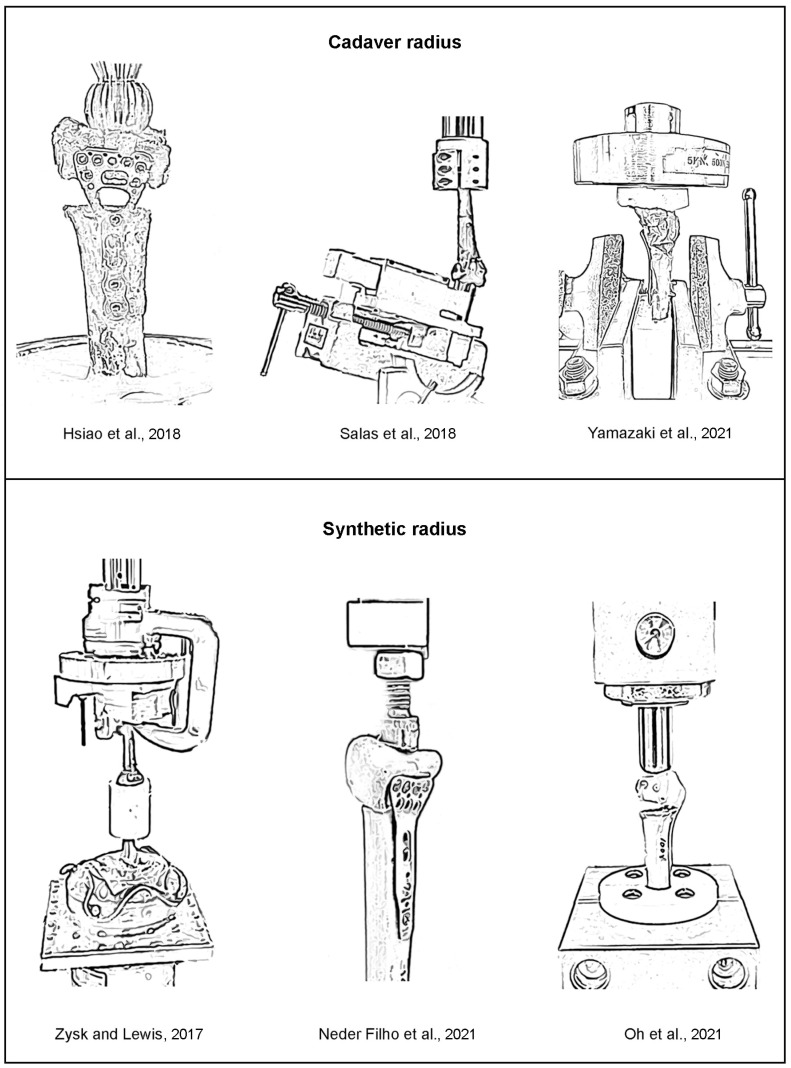
Previous biomechanical apparatus for the compressive tests of radii (Hsiao et al. [5]; Salas et al. [6]; Yamazaki et al. [7]; Zysk and Lewis [8]; Neder Filho et al. [9]; Oh et al. [10]).

The drawings of the biomechanical apparatus for the compressive tests of the previous works (i.e., cadaver radii [5,6,7] and synthetic radii [8,9,10]) are shown in Figure 5. Together with the descriptions of biomechanical apparatus given in the literature, it is speculated that the precise alignments between the applied compressive force at distal radius, the center line of radius, and the reaction compressive force at proximal radius of the previous biomechanical apparatus [5,6,7,8,9,10] may not be carefully considered. Therefore, the tested radii may be under the inconsistent combinations between compression, bending, and shear. Moreover, the applications of compressive force directly to the distal radii using the loading devices with simple geometries (e.g., sphere or flat shapes), instead of the more physiologically relevant intact wrist scenario, were observed in ref [5,6,9,10]. Mismatch between the contour of the loading device and the contour of the distal radius can cause the addition of bending and shear during biomechanical tests, which is likely to be inconsistent among repetitive biomechanical tests. Accordingly, the variability in the measured stiffnesses could be significantly high.

After the minimization of the above causes of inconsistency, the conditions of pure compression can be generated by the new biomechanical apparatus. The SD of the measured stiffness can be reduced, and the confidence in measured stiffnesses can be improved (especially when the stiffnesses of DRFs with various treatment methods are compared). Thus, the biomechanical apparatus and the experimental procedure could be recommended for the evaluation of compressive stiffness of synthetic radius.

For the real-world scenario (i.e., the cadaver radii with various geometries), the geometries of new biomechanical apparatus (i.e., the grip of fixture at the proximal radius and the loading device at the distal radius) can be designed to match with each individual cadaver radius. However, this is not economical and effective when many cadaver radii are tested. Accordingly, the biomechanical apparatus and the experimental procedure are more suitable for application with synthetic radii, where the geometry and material properties are successfully controlled by the manufacturer.

## 5. Conclusions

The biomechanical apparatus and the experimental procedure, which provides the conditions of pure compression, were proposed for the evaluation of radius stiffness. After biomechanical tests of the synthetic radius, it was confirmed that the conditions of pure compression and a low standard deviation of stiffness could be achieved. Accordingly, the biomechanical apparatus and the experimental procedure have proved to be a practical method for the evaluation of radius stiffness.

## Figures and Tables

**Table 1:** Compressive stiffnesses and deflections of the distal radii in the horizontal plane.

Repetition	Stiffness (N/mm)	Deflection (mm)			
		A—Ulnar	B—Dorsal	C—Radial	D—Volar
1	981.4	0.06	−0.01	0.07	−0.01
2	1016.2	0.06	−0.01	0.07	−0.01
3	990.9	0.06	−0.01	0.07	−0.01
4	1030.6	0.07	−0.01	0.07	−0.01
5	993.3	0.07	−0.01	0.07	−0.01
6	998.7	0.06	−0.01	0.07	−0.01
7	1015.3	0.06	−0.01	0.07	−0.01
8	1003.1	0.06	−0.01	0.07	−0.02
9	994.9	0.06	0.00	0.07	−0.01
10	1019.0	0.06	0.00	0.07	−0.01
Mean	1004.3	0.0620	−0.0080	0.0700	−0.0110
SD	±15.3	±0.0042	±0.0042	±0.0000	±0.0032

**Table 2:** Mean stiffnesses and standard deviations from various biomechanical tests of radii.

Research Work	Radius	Repetition (*n*)	Mean Stiffness (N/mm)	Standard Deviation (N/mm)
Hsiao et al. [5]	Cadaver distal radius with AO type C2 fracture fixed with a volar T-plate	6	426	±98
Salas et al. [6]	Cadaver distal radius with AO type 23-A3.2 extra-articular fracture fixed with an Acu-Loc2 volar distal radius plate	9	338	±112
Yamazaki et al. [7]	Cadaver distal radius with 10 mm dorsal wedge fixed with a VATCP	10	208	±56
Zysk and Lewis [8]	Intact synthetic distal radius	3	1372	±274
Neder Filho et al. [9]	Synthetic distal radius with 10 mm dorsal wedge fixed with a VLP	7	1170	±140
Oh et al. [10]	Synthetic distal radius with 10 mm dorsal wedge fixed with a VLP	2	595	±34 *
Present work	Intact synthetic radius	10	1004	±15

* Calculation from 2 tests.

## Data Availability

The raw and processed data generated during this study will be made available upon reasonable request.
